# Network of GRAS transcription factors in plant development, fruit ripening and stress responses

**DOI:** 10.1093/hr/uhad220

**Published:** 2023-09-27

**Authors:** Catarina Neves, Beatriz Ribeiro, Rute Amaro, Jesús Expósito, Jérôme Grimplet, Ana Margarida Fortes

**Affiliations:** BioISI–Biosystems and Integrative Sciences Institute, Faculty of Sciences, University of Lisbon, Campo Grande, 1749-016 Lisboa, Portugal; BioISI–Biosystems and Integrative Sciences Institute, Faculty of Sciences, University of Lisbon, Campo Grande, 1749-016 Lisboa, Portugal; BioISI–Biosystems and Integrative Sciences Institute, Faculty of Sciences, University of Lisbon, Campo Grande, 1749-016 Lisboa, Portugal; BioISI–Biosystems and Integrative Sciences Institute, Faculty of Sciences, University of Lisbon, Campo Grande, 1749-016 Lisboa, Portugal; Centro de Investigación y Tecnología Agroalimentaria de Aragón (CITA), Departamento de Ciencia Vegetal, Gobierno de Aragón, Avda. Montañana 930, 50059 Zaragoza, Spain; Instituto Agroalimentario de Aragón—IA2 (CITA-Universidad de Zaragoza), Calle Miguel Servet 177, 50013 Zaragoza, Spain; BioISI–Biosystems and Integrative Sciences Institute, Faculty of Sciences, University of Lisbon, Campo Grande, 1749-016 Lisboa, Portugal

## Abstract

The plant-specific family of GRAS transcription factors has been wide implicated in the regulation of transcriptional reprogramming associated with a diversity of biological functions ranging from plant development processes to stress responses. Functional analyses of GRAS transcription factors supported by *in silic*o structural and comparative analyses are emerging and clarifying the regulatory networks associated with their biological roles. In this review, a detailed analysis of GRAS proteins' structure and biochemical features as revealed by recent discoveries indicated how these characteristics may impact subcellular location, molecular mechanisms, and function. Nomenclature issues associated with GRAS classification into different subfamilies in diverse plant species even in the presence of robust genomic resources are discussed, in particular how it affects assumptions of biological function. Insights into the mechanisms driving evolution of this gene family and how genetic and epigenetic regulation of GRAS contributes to subfunctionalization are provided. Finally, this review debates challenges and future perspectives on the application of this complex but promising gene family for crop improvement to cope with challenges of environmental transition.

## Introduction

Transcription factors play an important regulatory role in several aspects of plant development and stress responses [1]. Plant-specific GRAS gene family has attracted interest due to their diverse biological functions and their widespread distribution across the plant kingdom. Structural and functional analyses *in silico* have characterized numerous GRAS genes in many plant species, from model plants to woody species including *Arabidopsis* [2, 3], rice [2, 3], melon [4], apple [5], grape [6], tomato [7], pepper [8], *Populus trichocarpa* [2], mosses and ferns [9], among others.

The GRAS gene family is named after the first three genes that were identified: *GIBBERELLIC ACID INSENSITIVE* (*GAI*), *REPRESSOR OF GA1* (*RGA*), and *SCARECROW* (*SCR*) [10]. Proteins from this family typically have a length of about 400 to 700 amino acids and have a very specific structure, with the proteins' carboxyl(C)-terminal possessing the conserved GRAS domain and the amino(N)-terminal being highly variable in both sequences and in length [11, 12]. Identifying the whole set of GRAS genes in each species allows us to group these genes in different subfamilies based on gene structure similarity by phylogenetic analysis, which not only reflects their evolutionary history but also works as a possible indicator of similar biological functions of orthologous genes [2, 3, 8, 13, 14]. Guo et al. [15] observed that the sequence similarity of the GRAS domain was highly conserved within the subfamily, indicating the possible conservation of the GRAS gene function.

GRAS genes and their respective proteins have been shown to play important roles during plant growth and development, fruit ripening, signalling, biotic and abiotic stress responses, among others [2, 3, 5–9, 11, 13, 14, 16, 17]. Members of the GRAS gene family are expressed in almost all plant tissues from the root to the fruits, with some of them exhibiting tissue-specific expression and/or differential expression under different stress conditions and developmental stages [6]. In this review, we summarize the most important findings of this important transcription factor family, debate future perspectives, and pose intriguing new questions.

### Structural and biochemical analyses of GRAS proteins

Transcription factors possess different motifs, binding affinities, and possibly functions within the C-terminal and N-terminal of the protein. The C-terminal of GRAS proteins composes the motifs leucine heptad repeat I (LHRI), VHIID, leucine heptad repeat II (LHRII), PFYRE, and the SAW [10, 12]. The LHR I motif possesses a nuclear localization signal (NLS) structure, while the VHIID motif is known to be the most conserved out of the five motifs, having appeared in all members of the GRAS protein family thus far [10, 18]. The LHRII structure is known as the leucine-rich region, containing a LXXLL structure [18, 19]. It has also been shown that the LHRI-VHIID-LHRII complex is involved in either protein–DNA or protein–protein interactions [10, 18]. Unfortunately, the function of the PFYRE and SAW motifs is not yet clear, although they seem to be necessary for the structural integrity of the GRAS domain or for protein function [18].

Regarding the N-terminal of the GRAS proteins, it has been demonstrated that it is important for the protein to perform specific functions, and this is due to the presence of intrinsically disordered regions (IDRs) [18, 20]. IDRs and their respective intrinsically disordered proteins (IDPs) are protein regions or functional proteins that do not possess, nor do they require a unique structure in order to perform their biological roles, challenging the classical “lock-and-key” model [21]. GRAS proteins are among the best characterized plant proteins known to contain IDRs [22]. Intrinsically disordered regions are important for several cellular functions, namely cell signalling and transcriptional regulation, and they seem to be more represented in eukaryotic proteomes [20–22]. This is due to the apparent fact that, even though IDRs or their respective can be found in all organisms from all life kingdoms and also in all the viral proteomes analysed thus far, bioinformatics research indicates that the abundance of disorder directly increases with the organism's complexity [21].

It is believed that IDRs are associated with the ability to adjust to changing environments [22]. These IDRs undergo several disorder-to-order transitions when they bind to other molecules, which bestow great functional diversity and flexibility to the GRAS proteins, and the conservation of these sequence parts in orthologous genes of different species suggests their involvement in protein functionality [13, 20, 22]. Furthermore, the N-terminal of GRAS proteins contains molecular recognition features (MoRFs) within IDRs, which may represent potential protein–protein binding sites [20]. Due to this, IDRs or the respective IDPs show a high selectivity in their interactions even if they possess low affinity with the other molecule [20, 22]. This allows the easy reversibility of their interactions, which is essential in several regulatory processes molecule [20, 22].

On a different note, short leucine-rich segments often act as nuclear export signals (NESs) [20]. However, the structural ambiguity of leucine-rich NES and the abundance of leucine-rich patterns in the proteome (e.g. thousands of leucine-rich repeats (LRR) resistance proteins in each genome) makes it harder to predict true NESs [20, 23]. It has been theorized that NES could be located within IDRs, since it is necessary that the nuclear-targeting signal is accessible for the efficient interaction with the proper NES receptor [20]. Hence, it is possible for certain GRAS proteins to possess NES motifs, gaining the ability to leave the nucleus, but this still requires further investigation [20].

Most GRAS proteins are thought to have a regulatory role, namely as transcription factors [10, 12]. This notion was mainly supported by the proteins' structural characteristics, specifically the homopolymeric stretches of amino acid residues and two LHR domains [12]. Both of which can be found in basic-leucine zipper (bZIP) protein family of transcription factors among others [12]. Furthermore, several GRAS genes also possess nuclear localization signals, and GRAS proteins were thought to bind directly to DNA [10, 24]. However, recent studies have shown that direct DNA binding may not be the only target of GRAS proteins nor the only mechanism in which they are involved in so many divergent processes [11]. Gene ontology (GO) enrichment analysis has shown other GRAS protein functions besides DNA binding, such as protein binding [15]. Additionally, even the assumption that GRAS protein interactions were confined to the nucleus has been put into question. Although most GRAS proteins activities are in the nucleus, some of the proteins with which they interact are in other cellular compartments as well, such as the plasma membrane and the cytoplasm [4, 15]. Furthermore, it has been demonstrated that one of the major ways for the GRAS gene family to regulate aspects of plant growth and development is by forming protein complexes [15]. Guo and co-workers [15] observed that different GRAS gene subfamilies possessed different types of interaction proteins, but mostly transcription factors. Two GRAS proteins, nodulation signalling pathway1 (NSP1) and NSP2, originate a DNA binding complex *in vivo* that promotes the efficient nodulation of *Medicago truncatula* [25]. Furthermore, Bi et al. [4] has predicted that the subcellular localization of the melon GRAS genes was mainly nuclear (75.68%), with a small portion of GRAS genes also being found in the cytoplasm (13.51%) and extracellularly (10.81%). Nevertheless, these assumptions are based on *in silico* data and still need to be ascertained *in vivo*.

Not all transcription factors are found in the nucleus; some are membrane-bound transcription factors (MTFs) and can be in dormant state [26]. Once an internal or external stimuli activates these MTFs, they are released from the membrane and transported to the nucleus [26]. Recently, it has also been discovered that transcription factors are new regulators of chloroplastidial gene expression [27]. The transcription factor NAC102 is localized in both the nucleus and chloroplasts in *Arabidopsis*, directly interacts with chloroplast RNA polymerases and functions as a repressor, since the overexpression of *NAC102* leads to reduced chloroplastidial gene expression and chlorophyll content [27]. With this new evidence, together with the possibility of the existence of a NES motif within the N-terminal of GRAS genes, it is not unlikely that GRAS proteins can act in other cellular compartments as well.

### GRAS subfamilies and associated nomenclature

Due to the increase of genomic resources regarding the GRAS gene family in many species, it was observed that the conservation of certain specific sequences could be grouped within different subfamilies. Tian et al. [3] have firstly separated the GRAS gene family in DELLA, HAM, LISCL, PAT1, LS, SCR, SHR, and SCL3 subfamilies. DELLA proteins are easily distinguished from other GRAS proteins due to the presence of a DELLA domain in the N-terminal region of the protein, and these proteins are known to be involved in the regulation of the gibberellin acids (GAs) signalling mechanisms [3, 8]. Genes from the HAM subfamily are necessary for the maintenance of shoot and root indeterminacy; the first gene belonging to this family *HAIRY MERISTEM* was identified in *Petunia* [28, 29]. The LISCL subfamily is responsible for the transcriptional regulation of microsporogenesis in *Lilium longiflorum*, while genes belonging to the PAT1 subfamily are known to be involved in phytochrome A signal transduction in *Arabidopsis thaliana* [30, 31]. The LS subfamily includes genes that are responsible for axillary meristem initiation and lateral shoot formation, with the first gene *LATERAL SUPPRESSOR* being identified in tomato [32]. Genes from both SCR and SHR subfamilies are involved in the regulation of root growth, and are known to interact with each other [10, 33]. Similarly, the SCL3 subfamily, whose first identified gene was *SCARECROW-LIKE 3*, is also involved in root development and growth, namely in endodermal specification [10].

However, the classification and subdivision of the GRAS family differ slightly based on phylogenetic relationships, depending on the amount and variety of species used for the study [14]. Tian et al. [3] only used *Arabidopsis* and rice originally, to understand how the GRAS family evolved in monocot and dicot plants. Grimplet et al. [6] conducted a phylogenetic study with 16 different plant species, which resulted in the discovery of five new subfamilies: SCL26, GRAS8, with namesake *Arabidopsis* genes previously not identified in a subfamily, and GRASV1, GRASV2, and GRASV3 only described in dicots. Since the first subfamily division, the number of subfamilies has increased, with each plant species possessing GRAS genes included within 8 to 17 different subfamilies (Table 1 and Supplementary Table S1). The GRAS gene family possesses a total of 19 identified subfamilies [15]. Subfamilies once thought to be species-specific, nowadays are shared between different species. For instance, the Pt20 subfamily was initially thought to be exclusive of *Populus* [2] but later it was identified in tomato [7]. The division of the GRAS gene family in subfamilies will facilitate the future identification of orthologue genes in different plant species [13]. Even though gene orthology is not a direct indicator of function conservation, orthologous genes are still the best candidates for functional transfer between plant species [13].

**Table 1:** Diversity of nomenclature associated with GRAS gene subfamilies in different plant species

Species	Name of the subfamilies	References
*Arabidopsis thaliana* $ \raisebox{-2pt}{\includegraphics{\bwartpath uhad220fx1}} $	LISCL, AtPAT1, AtSCL3, DELLA, AtSCR, AtSHR, AtLAS, HAM, AtSCL4/7, DLT	2
	LISCL, PAT1, SCL3, DELLA, SCR, SHR, LS, HAM	3
	LISCL, PAT1, SCL3, DELLA, SCR, SHR, LAS, HAM, SCL4/7, DLT	9
	LISCL, PAT, SCL3, DELLA, SCR, SHR, LS, HAM, SCL4/7, DLT, SCL32, NSP1, NSP2	13
*Populus trichocarpa* $ \raisebox{-2pt}{\includegraphics{\bwartpath uhad220fx2}} $	LISCL, AtPAT1, AtSCL3, DELLA, AtSCR, AtSHR, AtLAS, HAM AtSCL4/7, DLT, Os4, Os19, Pt20	2
*Oryza sativa* $ \raisebox{-2pt}{\includegraphics{\bwartpath uhad220fx3}} $	LISCL, AtPAT1, AtSCL3, DELLA, AtSCR, AtSHR, AtLAS, HAM, AtSCL4/7, DLT, Os4, Os19	2
	LISCL, PAT1, SCL3, DELLA, SCR, SHR, LS, HAM	3
	LISCL, PAT1, SCL3, DELLA, SCR, SHR, LAS, HAM, SCL4/7, DLT, Os4, Os19, Os43	9
	LISCL, PAT, SCL3, DELLA, SCR, SHR, LS, HAM, SCL4/7, DLT, SCL32, NSP1, NSP2, RAD1, RAM1, SCLA	13
*Cucumis melo* $ \raisebox{-2pt}{\includegraphics{\bwartpath uhad220fx4}} $	PAT1, SCL3/28, DELLA, SCR, SHR, LAS, HAM SCL4/7, SCL9	4
*Malus domestica* $ \raisebox{-2pt}{\includegraphics{\bwartpath uhad220fx5}} $	LISCL, PAT1, SCL, DELLA, SCR, SHR, LS, HAM	5
*Vitis vinifera* $ \raisebox{-2pt}{\includegraphics{\bwartpath uhad220fx6}} $	LISCL, PAT, SCL3, DELLA, SCR, SHR, LS, HAM, SCL26, GRAS8, GRASV1, GRASV2, GRASV3	6
	LISCL, PAT, SCL3, DELLA, SCR, SHR, LS, HAM, SCL4/7, DLT, SCL32, NSP1, NSP2, RAD1, RAM1, SCLA, SCLB	13
*Brassica napus* $ \raisebox{-2pt}{\includegraphics{\bwartpath uhad220fx7}} $	LISCL, PAT, SCL3, DELLA, SCR, SHR, LS, HAM, SCL4/7, DLT, SCL32, NSP1, NSP2	15
*Solanum lycopersicum* $ \raisebox{-2pt}{\includegraphics{\bwartpath uhad220fx8}} $	AtPAT1, AtSCL3, DELLA, AtSCR, AtSHR, AtLAS, HAM, AtSCL4/7, Os4, Os19, Pt20, AtSCL9, AtSCL28	7
*Capsicum annuum* $ \raisebox{-2pt}{\includegraphics{\bwartpath uhad220fx9}} $	LISCL, PAT1, SCL3, DELLA, SCR, SHR, LAS, HAM, DLT, Ca_GRAS	8
*Triticum aestivum* $ \raisebox{-2pt}{\includegraphics{\bwartpath uhad220fx10}} $	LISCL, PAT1, SCL3, DELLA, SCR, SHR, LAS, HAM, SCL4/7, DLT, Os4, Os19	14
*Brachypodium distachyon* $ \raisebox{-2pt}{\includegraphics{\bwartpath uhad220fx11}} $	LISCL, PAT1, SCL3, DELLA, SCR, SHR, LAS, HAM, SCL4/7, DLT	34
*Lagenaria siceraria* $ \raisebox{-2pt}{\includegraphics{\bwartpath uhad220fx12}} $	LISCL, PAT, SCL3, DELLA, SCR, SHR, LS, HAM, SCL4/7, DLT, SCL32, NSP1, NSP2, RAD, RAM1, SCLB	35
*Hordeum vulgare* $ \raisebox{-2pt}{\includegraphics{\bwartpath uhad220fx13}} $	LISCL, PAT1, SCL3, DELLA, SCR, SHR, LAS, HAM, SCL4/7, DLT, Os19, Os43	36
*Solanum tuberosum* $ \raisebox{-2pt}{\includegraphics{\bwartpath uhad220fx14}} $	LISCL, PAT1, SCL3, DELLA, SCR, SHR, LS, HAM	18
*Glycine max* $ \raisebox{-2pt}{\includegraphics{\bwartpath uhad220fx15}} $	LISCL, PAT1, SCL3, DELLA, SCR, SHR, LAS, HAM, SCL4/7	17
	LISCL, AtPAT1, AtSCL3, DELLA, AtSCR, AtSHR, HAM, AtSCL4/7, DLT, Os4, Os19	37
*Panax ginseng* $ \raisebox{-2pt}{\includegraphics{\bwartpath uhad220fx16}} $	LISCL, PAT, SCL3, DELLA, SCR, SHR, HAM, SCL4/7, DLT, NSP1, NSP2, RAM1, PG1	38
*Ricinus communis* $ \raisebox{-2pt}{\includegraphics{\bwartpath uhad220fx17}} $	LISCL, PAT1, SCL3, DELLA, SCR, SHR, HAM, SCL4/7, DLT, Os4, Os19, Os43, Rc_GRAS	39
*Dendrobium catenatum* $ \raisebox{-2pt}{\includegraphics{\bwartpath uhad220fx18}} $	LISCL, AtPAT1, AtSCL3, DELLA, AtSCR, AtSHR, AtLAS, HAM, AtSCL4/7, DLT, Unknown subfamily	40
*Gossypium hirsutum* $ \raisebox{-2pt}{\includegraphics{\bwartpath uhad220fx19}} $	LISCL, PAT1, SCL3, DELLA, SCR, SHR, LAS, HAM, SCL4/7, DLT, Os4, Os19, Os43, G_GRAS	9
*Gossypium arboreum* $ \raisebox{-2pt}{\includegraphics{\bwartpath uhad220fx20}} $	LISCL, PAT1, SCL3, DELLA, SCR, SHR, LAS, HAM, SCL4/7, DLT, Os4, Os43, G_GRAS	9
*Gossypium raimondii* $ \raisebox{-2pt}{\includegraphics{\bwartpath uhad220fx21}} $	LISCL, PAT1, SCL3, DELLA, SCR, SHR, LAS, HAM, SCL4/7, DLT, Os4, Os43, G_GRAS	9
*Physcomitrium patens* $ \raisebox{-2pt}{\includegraphics{\bwartpath uhad220fx22}} $	LISCL, PAT1, SCL3, DELLA, SCR, SHR, LAS, HAM, SCL4/7, DLT, Os4, Os19, Os43, G_GRAS, PSG	9
*Selaginella moellendorffii* $ \raisebox{-2pt}{\includegraphics{\bwartpath uhad220fx23}} $	LISCL, PAT1, SCL3, DELLA, SCR, SHR, LAS, HAM, SCL4/7, DLT, Os4, Os19, Os43, G_GRAS, PSG	9
*Amborella trichopoda* $ \raisebox{-2pt}{\includegraphics{\bwartpath uhad220fx24}} $	LISCL, PAT, SCL3, DELLA, SCR, SHR, LS, HAM, SCL4/7, DLT, SCL32, NSP1, NSP2, RAD1, RAM1, SCLA, SCLB	13
*Phoenix dactylifera* $ \raisebox{-2pt}{\includegraphics{\bwartpath uhad220fx25}} $	LISCL, PAT, SCL3, DELLA, SCR, SHR, LS, HAM, SCL4/7, DLT, SCL32, NSP1, NSP2, RAD1, RAM1, SCLA, SCLB	13
*Musa acuminata* $ \raisebox{-2pt}{\includegraphics{\bwartpath uhad220fx26}} $	LISCL, PAT, SCL3, DELLA, SCR, SHR, LS, HAM, SCL4/7, DLT, SCL32, NSP1, NSP2, RAD1, RAM1, SCLA	13
*Theobroma cacao* $ \raisebox{-2pt}{\includegraphics{\bwartpath uhad220fx27}} $	LISCL, PAT, SCL3, DELLA, SCR, SHR, LS, HAM, SCL4/7, DLT, SCL32, NSP1, NSP2, RAD1, RAM1, SCLA, SCLB	13
*Coffea canephora* $ \raisebox{-2pt}{\includegraphics{\bwartpath uhad220fx28}} $	LISCL, PAT, SCL3, DELLA, SCR, SHR, LS, HAM, SCL4/7, DLT, SCL32, NSP1, NSP2, RAD1, RAM1, SCLA, SCLB	13
*Fragaria vesca* $ \raisebox{-2pt}{\includegraphics{\bwartpath uhad220fx29}} $	LISCL, PAT1, SCL3, DELLA, SCR, SHR, LAS, HAM, SCL4/7, DLT, Os4, Os19, Os43, Fve39	41
*Prunus mume* $ \raisebox{-2pt}{\includegraphics{\bwartpath uhad220fx30}} $	LISCL, PAT1, SCL3, DELLA, SCR, SHR, LS, HAM, Group IX, Group X, Group XI	42

One of the main issues regarding the study of any gene family is the lack of a unifying database and nomenclature system. In this regard for grapevine, a Super-Nomenclature Committee for Grape Gene Annotation (sNCGGA) was created to develop a standard nomenclature for locus identifiers and also to create guidelines for a gene naming system for grapevine genomics [43]. One of the recommendations points to check previous works for synonyms and to preserve them as synonym, even if a new name needs to be attributed; this was applied to the GRAS gene family in grapevine [6, 43]. This helps avoiding any confusion regarding the identity of a studied gene or subfamily. Regarding the nomenclature of the GRAS subfamilies, the lack of consensus is still a complex issue (Table 1). For example, the PAT subfamily is often referred to in literature as both PAT [6, 13, 15, 35, 38] and PAT1 [2–5, 7–9, 14, 17, 18, 34, 36, 37, 39–42]. Furthermore, the LS subfamily has different names depending on the species studied. For instance, Tian et al. [3] names it LS subfamily together with several authors [5, 6, 13, 15, 18, 35, 42]. However, other authors refer to the same subfamily as LAS [2, 4, 7–9, 14, 34, 36, 37, 40, 41]. This type of nomenclature inconsistencies originates confusion regarding the subfamily being studied. Therefore, establishment of clear nomenclature rules should be a priority.

### Genetic and epigenetic regulation of GRAS transcription factors

Transcription factors have been described to be regulated by both genetic and epigenetic mechanisms. Genes from certain GRAS subfamilies, such as *SCARECROW* (*SCR*) and *SHORTROOT* (*SHR*), are known to regulate each other's expression levels [33]. The SHR protein is essential for the endodermis specification in the *Arabidopsis* root, and its movement is limited to the first cell layer due to the action of SCR, which sequesters SHR in the nucleus through protein–protein interactions and a safeguard mechanism which relies on a SHR/SCR-dependent positive feedback loop for the transcription of *SCR* [33].

Epigenetic modifications have also been shown to play a role in regulation of *GRAS* genes' expression in addition to genetic factors, such as *cis-*regulatory elements [14]. The expansion of GRAS gene family in wheat (*Triticum aestivum*) was reported to be mainly due to tandem and segmental duplications that may lead to subfunctionalization and increased adaptation to environmental transitions [14]. The expression of some of these *GRAS* genes in wheat was shown to be affected by histone modifications or through DNA methylation. These epigenetic mechanisms contributed for their subfunctionalization and may generate evolutionary novelty in plant genomes [14].

MicroRNAs (miRNAs) also play an important role in regulating gene expression [44]. These noncoding RNAs are known to perform the degradation of target mRNAs [11]. Recently, it has been reported that several GRAS members are regulated by miRNAs, especially by miRNA171 [24, 45]. In fact, most GRAS genes belonging to the HAM subfamily seem to be regulated by this specific miRNA [15]. Genes from the HAM subfamily that possess complementarity with miRNA171 can be found in several plant species, including *Brassica napus* [15], *Solanum lycopersicum* [7], *Ricinus communis* [39], *Gossypium hirsutum* [9], among others. In rice and barley, overexpression of miRNA osa-miR171 led to decreased expression of at least one HAM gene [46] and prolongation of vegetative state, through the maintenance of SAM indeterminacy [46, 47]. These studies indicate that regulatory mechanisms behind gene expression of GRAS family may be largely not only genetic but also epigenetic.

### GRAS diversity and evolution across plant species

**Figure 1:**
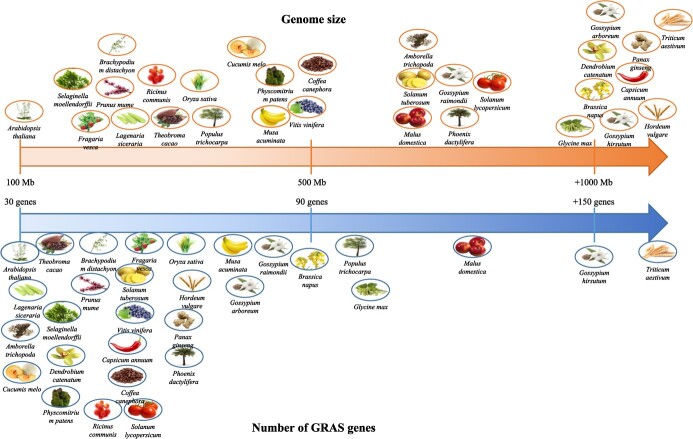
Schematic representation of the genome size and number of GRAS genes in different plant species. The upper part of the figure shows the genome size of different species in relation to each other while the lower part shows the number of GRAS genes found in each of those species. Additional information is available in Supplementary Table S1.

The number of GRAS genes seems to not have a direct correlation with genome size (Fig. 1). It also seems not to be dependent on whether plant species are dicotyledons or monocotyledons, nor woody or herbal plants [42]. In fact, it seems that genome duplications are the main force driving the expansion of the GRAS gene family [42]. *M. domestica* that had a recent whole-genome duplication (WGD) possesses one of the highest numbers of GRAS genes. Regarding distribution along chromosomes, GRAS genes are unevenly distributed. For example, in grapevine, the highest number is found in only 2 of the 19 chromosomes due to gene repeats that belonged to the same subfamily; these repeats may have arisen from ancestral polyploidization events [6].

Most GRAS genes do not possess introns. This is uncommon in eukaryotic genomes with intronless genes arising either by phenomena of horizontal gene transfer, retroposition of intron-containing genes, or duplication of intronless genes [48]. It is believed that the origin of the plant GRAS genes lies in their horizontal gene transfer from ancient prokaryotic soil bacteria genomes followed by several duplication events in flowering plants, explaining the abundance of intronless genes [2, 16, 49]. The GRAS gene family has greatly expanded in the fast-growing woody tree species, which accentuates their importance in plant growth and development [2]. It is believed that this gene family first appeared or at least significantly expanded approximately 580 million years ago in the common ancestor of Zygnematophyceae, a group of streptophyte algae, and embryophytes, a sister group of land plants (Bryophytes), both of which share the same subaerial/terrestrial habitat [16]. Until now, no GRAS genes have been found in other algae groups [9, 15, 16].

It is possible that three duplications of the GRAS genes occurred in the common ancestor of Zygnematophyceae and embryophytes, with further genome duplications and diversified selection occurring after the evolutionary split [3, 16, 50]. Overtime, some GRAS genes have developed a different exon–intron structure, which suggests that they likely gained new functions to better adapt to a specific environment [9, 37]. The HAM subfamily is one of the most ancient subfamilies, and since the target sequence for the miRNA171 is highly conserved in different plant species, it seems that the regulation mechanism of *HAM* genes by miRNA171 was formed in ancestral species and was conserved in land plants [15]. In angiosperms, the identification of 29 orthologue groups is an indicator of the large expansion and functional diversification of the GRAS gene family [13]. The loss of members or even the complete loss of a certain orthologue group is an indicator that in those species this loss may be compensated by another close orthologue group, meaning that members of the GRAS gene family of close orthologue groups remain redundant to a certain extent [50]. In the work of Grimplet et al. [6], for example, no grapevine GRAS gene homology was found within the LISCL subfamily in strawberry, suggesting that it may be absent in strawberry.

Since GRAS family first appeared in plants, several subfamilies have been lost or regained in a somewhat direct correlation with the habitat occupied by the species [16]. Interestingly, after terrestrialization the plant species that reverted to an aquatic environment lost several GRAS genes subfamilies and other genes associated with a terrestrial lifestyle, such as genes involved in the arbuscular mycorrhizal symbiosis [16].

### Involvement of GRAS in plant growth and development

The *GRAS* gene family is differentially expressed across different plant tissues putting in evidence their diverse roles in plant development [11, 24].

#### Root apical meristem maintenance and root development

One of the first GRAS mutants to be identified was the *SCARECROW* (*SCR*) mutant which presents an abnormal root formation phenotype [51]. In this work, it was shown that SCR regulates the asymmetric cell division involved in the radial organization of the Arabidopsis root [51].The SHORT-ROOT (SHR) also plays an essential role in root development [52]. SHR moves from the stele into the root endodermis while SCR is involved in nuclear accumulation of SHR. Mutant analyses showed that SHR movement from the stele is essential for normal patterning of the root in Arabidopsis [52]. More recently, SCR was shown to coordinate cell elongation, endodermal differentiation, redox homeostasis, and oxidative stress response in the root. Additionally, SCR acts independently of SHR, but these two transcription factors still function similarly in other aspects of root growth and development [53].

**Figure 2:**
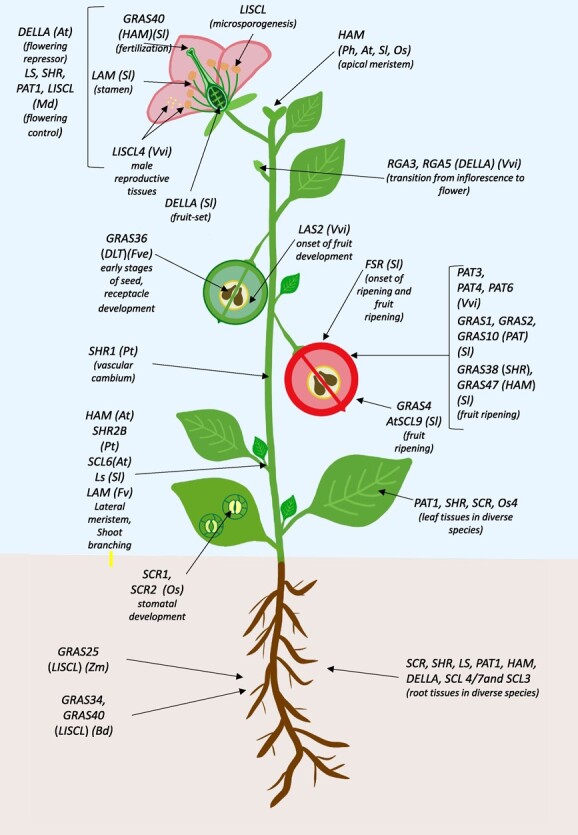
Schematic representation of GRAS sub-families and GRAS genes' expression in different plant tissues. Involvement of GRAS in diverse developmental processes is highlighted. Species where the studies were conducted are shown in parenthesis: *At, Arabidopsis thaliana; Bp*, *Brachypodium distachyon; Fve, Fragaria vesca; Md, Malus domestica; Os, Oryza sativa; Sl, Solanum lycopersicum; Ph, Petunia hybrida; Vvi, Vitis Vinifera; Zm, Zea mays*. References are mentioned in the main text.

Other studies involving transcriptomics also indicate an important role of GRAS in root development. Members of SCR, SHR, and LS were highly or specifically expressed in the root of *B. napus* while other diverse subfamilies could also be represented such as LISCL [15]. In *Zea mays* L., the *ZmGRAS25* which belongs to this sub-family was highly expressed in primary root tissue (Fig. 2) [54]. In *Prunus mume*, 11 *GRAS* genes from the PAT1, SHR, SCR, HAM subfamilies were also expressed abundantly in the root [42], whereas in Bottle gourd (*Lagenaria siceraria*) members of DELLA, PAT, SCL 4/7, HAM, and SCL3 subfamilies were modulated [35]. In *Brachypodium distachyon, GRAS* were found expressed in a broad range of tissues, nevertheless certain genes showed tissue specificity, such as *BdGRAS34* and *BdGRAS40* (LISCL sub-family), which were highly expressed in root [34, 55]. In *G. hirsutum*, *DELLA* genes showed expression patterns that indicate that they might be involved in regulating root elongation [9], while in rice, members of the PAT1 subfamily may be related to root–shoot transition according to their expression patterns [56].

These data indicate that, depending on the plant species, different subfamilies may be involved in root growth and development. However, functional characterization is required to ascertain the roles played by GRAS genes. Previously, RGA1 (DELLA) was shown to be a gibberellin acid signalling repressor involved in a complex acting on root growth, among other developmental processes [57]. A member of another GRAS subfamily, PAT1, was shown to form a complex with ETHYLENE RESPONSE FACTOR115 (ERF115–PAT1) that is essential for the recovery of the root meristem upon root tip excision, therefore enabling regeneration competence [58]. These studies highlight the interaction of GRAS transcription factors and hormonal signalling, which plays an important role in plant development.

#### Shoot apical meristem maintenance and shoot development

Formation of new plant organs depends on the maintenance of the Shoot Apical Meristem (SAM) [1]. Members of the HAM sub-family, *LOM1* or *LOM2*, are required for SAM maintenance in Arabidopsis [59]. The *lom (LOST MERISTEMS)* mutants showed arrested meristems characterized by an over-proliferation of meristematic cells and no polar organization [59].

The *Petunia hybrida HAIRY MERISTEM* (*HAM*) promotes shoot indeterminacy [60]. In the *ham* mutant, shoot meristems differentiate post embryonically as extensions of the bounding area of the stem [29]. HAM acts in similar ways to the TERMINATOR (PhWUSCHEL). All *ham* mutants aborted organ formation during vegetative growth before transition to flowering indicating that HAM signals cell fate in the shoot apex [29]. Furthermore, *Atham1,2,3* mutants presented abnormal shoot phyllotaxis and lateral organ formation and changed meristem morphology [60]. Additionally, overexpression of *SlGRAS24* (HAM sub-family) led to disturbance of GA and auxin signalling and dwarfism in tomato [61], which may indicate that editing of this gene may lead to taller plants. Tomato plants overexpressing *SlGRAS24* also showed short primary roots with less lateral roots and more lateral branches, indicating that *HAM* genes may regulate endogenous GA/auxin balance in the diverse meristems [61].

Indeed, *GRAS* domain HAIRY MERISTEM (HAM) family members play essential roles in regulation of shoot meristem activity in several monocots and dicots. In rice, it was found that the maintenance of SAM indeterminacy is controlled by *OsHAM1*, *OsHAM2*, *OsHAM3*, and *OsHAM4* which are targeted by osa-miR171c to regulate the onset of phase transition from vegetative to reproductive development [46]. On the other hand, transgenic maize plants failed to fully rescue the meristem defects in the *ham123* mutant and the *CLV3* (*CLAVATA3*) expression pattern was different from the wild type [62]. Zhou et al. [63] established a model where HAM proteins were responsible for preventing WUS-mediated induction of *CLV3* expression. More recently, it was found that HAM family members have overlapping and distinct roles in the control of *CLV3* patterning [64]. *WUS* and *CLV3* are exclusively expressed in a few SAM cells in contrast to *HAM1* and *HAM2*. This suggests that *HAM1/2* have broader roles that are independent of *CLV3* and *WUS* [64]. Additionally, GRAS were shown to be involved in not only primary meristem but also in secondary meristem regulation. Indeed, in poplar, *PtSHR2B* appears to be involved in lateral meristem functioning and *PtSHR2B* appears to play a role alongside *PtSHR1* in regulating vascular cambium activity [65].

Interestingly, a study on the HAM subfamily found that it originated after the divergence of streptophyte algae and may be represented in almost all land plant species [66]. Also, a *HAM* gene was duplicated in the common ancestor of angiosperms after the divergence of gymnosperms and angiosperms. In fact, this study indicated that the regulation of *HAM* is an ancestral trait [66].

#### Lateral organ formation and leaf organogenesis

Transcriptional remodelling in the SAM with the involvement of several subfamilies of GRAS leads to organogenesis of new primordia. In *A. thaliana SCARECROW-LIKE6-II* (*SCL6-II*), *SCL6-III*, and *SCL6-IV* were shown to be targets of the microRNA171c (miR171c) which negatively controls shoot branching [67]. The loss of function triple mutants showed that these genes are transcriptional activators involved in this developmental process (Fig. 2) [67]. The initiation of axillary meristems in tomato is reduced but not abolished in the *lateral suppressor* (*ls*) mutant, denoting that this GRAS transcription factor is not essential for axillary meristem formation [32]. However, in woodland strawberry (*Fragaria vesca*), the *loss of axillary meristems (lam)* mutant presented a loss of axillary meristems and reduced number of branch crowns and runners [68]. This phenotype was caused by a failure in axillary meristem initiation [68].

In sweet potato *GRAS*, *ItfGRAS12* (SHR), *ItfGRAS45* (SCR), and *ItfGRAS59* (PAT1) were more expressed in the leaf and stem than in the other tissues; these three genes were suggested to be putative regulators of SAM and axillary meristem differentiation [69]. On the other hand, in woodland strawberry only *FveGRAS33* belonging to the Os4 GRAS sub-family, showed high expression in leaves [41]. The Os4 sub-family is not present in other species, which indicates a lineage-specific gene diversity and specialization.

In wild-type tomato plants *SlGRAS10* (PAT1) was also highly expressed in leaves; the length and width of leaves of some *SlGRAS10*-RNAi lines was remarkably decreased together with reduced plant height and internode length [70]. In other studies in tomato, *PAT1* genes exhibited high expression in both newly developed and mature leaves [71], while *SlGRAS26* (AtSHR) and *SlGRAS37*(AtSCR) showed particularly high levels of expression in leaf and bud [7]. Also in tomato, the *procera* mutation caused by a *DELLA* gene leads to changes in the plant architecture through abnormal branching with the *pro* mutant exhibiting an elongated phenotype [72].

Interestingly, loss of function mutants for the redundant rice genes *OsSCR1* and *OsSCR2* presented leaves lacking stomata (Fig. 2). These genes act upstream of the *OsMUTE* and *OsFAMA* genes in stomatal development, and *SCR* may regulate their initiation [73].

From the above, it is clear that lateral organ formation and leaf organogenesis may involve different GRAS subfamilies depending on the species or even varieties.

#### Flower, embryo, and seed development

GRAS transcription factors have been reported to play a role in flower, embryo, and seed development. In Arabidopsis, a pentuple *DELLA* mutant showed earlier flowering indicating that these transcription factors act as repressors of flowering [74]. The observation that the early flowering ga1–3 gai-t6 rga-t2 rgl1–1 rgl2–1 mutant exhibits increased *FLOWERING LOCUS T (FT)* and *SQUAMOSA PROMOTER BINDING PROTEIN-LIKE 3* (*SPL3)* expression indicates that DELLA proteins act by repressing *FT* and *SPL3* which are involved in the regulation of the onset of flowering [74].

In tomato 16 of 40 *SlGRAS* genes exhibited higher expression in stamen and 12 genes were more abundant in ovary tissues; this is indicative of the functional specialization of the *GRAS* gene family members in tomato floral organs [7]. Additionally, lines overexpressing *SlGRAS24* (HAM subfamily) showed up to a 75% decrease in fruit set and smaller fruits with fewer seeds. In accordance, flower transcriptome at anthesis showed significant changes in the expression of genes involved in pollen development and hormonal signalling [61]. In apple, expression of *MdGRAS6* (LS), *MdGRAS26* (SHR), *MdGRAS44* (PAT1), *MdGRAS53*, *MdGRAS107*, *MdGRAS122* (DELLA), and *MdGRAS64* (LISCL) was significantly modulated in the bud indicating their role in flowering. During flower induction under hormonal treatments, the expression patterns of LS, SHR, SCL, PAT1, LISCL, and DELLA members indicated they were also involved in gibberellic acid, 6-benzyladenine, and/or sugar-mediated flowering in apple trees [5].

The overexpression of *SlGRAS40* also from the HAM subfamily in tomato led to smaller fruit, disruption of fertilization with a reduced fruit set ratio, and reduced number of seeds with decreased fruit weight and production. This disturbed fertilization was linked to eventual disruption in auxin and gibberellin metabolisms in pollinated ovaries [75].

The previously mentioned *procera* (*pro*) mutant (*SlDELLA)* presents elongation of style, which prevents self-pollination, increased number of flowers, and seedless fruits (parthenocarpic phenotype) [76]. However, the *pro* mutants when manually pollinated recovered the normal seed number [76]. Also, a tomato antisense *SlDELLA* (Fig. 2) line presented smaller and seedless fruits with an elongated shape [77]. However, when hand pollinated the antisense lines restored wild-type fruit phenotype. This indicates that fertilization-associated *SlDELLA*-independent signals are operational in ovary–fruit transitions. Indeed, it was shown that *SlDELLA* controls fruit set during anthesis arrest and regulates pericarp cell expansion in the following stages. *SlDELLA* gene seems to operate as a growth repressor during fruit development, and manipulation of this gene leads to morphological changes in the style that hinder normal fertilization [77].

Other GRAS subfamilies are also involved in flower development and fertilization-associated processes. The *LAM* gene was found to be expressed in all floral meristematic tissue and young floral organs in strawberry [68]. The study of *lam* mutants showed that LAM was crucial for stamen initiation, but this role may be species-specific [68]. In grape, *VviLISCL4* was predominantly expressed in male reproductive tissues, stamen, and pollen, whereas *VviRGA3* and *VviRGA5* were up-regulated during fruit set (Fig. 2) and may regulate the transition from inflorescence to flower [6]. Morohashi et al. [31] identified a *LlSCL* gene (*L. longiflorum Scarecrow-like*), which was mainly expressed at the premeiotic phase within anthers (Fig. 2). Their results suggest that LlSCL functions as a co-activator in triggering gene expression associated with regulation of microsporogenesis.

#### Fruit development and ripening

Fruit development and ripening are strictly regulated developmental processes. Huang et al. [7] noted in tomato (climacteric fruit) a higher abundance of *GRAS* transcripts in immature fruits than mature fruits, but some genes, such as *SlGRAS38* (AtSHR), *SlGRAS35,* and *SlGRAS47* (HAM), display a strong and tissue-specific expression during fruit ripening (Fig. 2). Functional analysis of *SlFSR (SlGRAS38)* showed that its silencing greatly prolonged tomato shelf-life with decreased activity of enzymes involved in cell wall degradation [78]. The overexpression of *SlFSR* in *rin* lines led to a similar inhibited ripening to that of the *rin* mutant and had comparable content of ethylene and carotenoids. The authors hypothesized that RIN might target *SlFSR* which then would regulate cell wall metabolism (Fig. 2) [78]. Also in tomato Liu et al. [79] found that *SlGRAS4* (AtSCL9) was induced during fruit ripening and its expression is modulated by ethylene. The overexpression of *SlGRAS4* accelerated fruit ripening (Fig. 2), but *SlGRAS4*-RNAi fruits presented normal ripening which may be caused by other ripening regulators that function in a complementary manner [79]. In *P. mume*, which also exhibits climacteric fruit ripening, the expression pattern of *PmGRAS15* (*HAM*) and *PmGRAS42* (unnamed *P. mume*-specific subfamily) indicated that both transcription factors have important functions during later stages of fruit development [42].

*GRAS* genes are also involved in fruit development in the non-climacteric strawberry. While *FveGRAS36* gene (DLT subfamily) was mainly expressed during the early stages of seed and receptacle development, *FveGRAS54* (*PAT1* subfamily) was predominantly expressed in ripening fruit (Fig. 2) [41]. On the other hand, expression of all *SHR* and *SCL3* family genes was insignificant and decreased from the immature stage to the ripening stage; this was suggested to be due to ethylene not being required for ripening in strawberry [41]. In the non-climacteric grapevine fruit, *VViPAT3*, *VviPAT4*, and *VViPAT6* (Fig. 2) were highly in ripe fruit but patterns may change depending on the cultivar [6]. The same holds true for their tomato orthologues, *SlGRAS1*, *SlGRAS2*, and *SlGRAS10* [6]. *VviLAS2* (Fig. 2) was more expressed in the beginning of fruit development and *VviLAS1* in mature berries, indicating a putative role of this subfamily in fruit development and ripening [6].

Further functional analyses are necessary in climacteric and non-climacteric fruit crops in order to disclose how different hormonal metabolisms may affect involvement of GRAS members in fruit ripening.

### Involvement of GRAS in abiotic stress responses

Abiotic stresses, such as salinity, drought, heat, cold, nutrient deficiency, hypoxia, UV-radiation, and heavy metal toxicity, negatively affect plant growth, development, and productivity [80]. The GRAS gene family are key signalling components in the process of responding to abiotic stresses [81] and have been shown to induce tolerance to these stresses in various plant species by affecting the expression of various stress-related genes (Table 2, Supplementary Fig. 1).

**Table 2:** Involvement of GRAS genes in response to multiple abiotic stresses as revealed by homologous and heterologous functional analyses. Abbreviations: *Ca, Capsicum annum; Sl, Solanum lycopersicum; Pe, Populus euphratica; Ta, Triticum aestivum; Va, Vitis amurensis*

**Species**	**GRAS gene**	**GRAS subfamily**	**Function**	**Target genes/ Pathways**	**Reference**
					
*Arabidopsis thaliana* $ \raisebox{-2pt}{\includegraphics{\bwartpath uhad220fx31}} $	*PeSCL7*	SCL4/7	Enhanced tolerance to Salinity/ Drought	Elevated transcript levels and activity of stress responsive enzymes (α-amylase and superoxide dismutase) in *A.thaliana* overexpressing *PeSCL7*	82
*A. thaliana* $ \raisebox{-2pt}{\includegraphics{\bwartpath uhad220fx32}} $	*VaPAT1*	PAT1	Enhanced tolerance to Salinity/ Drought/ Cold	Higher expression of stress-related genes (*AtSIZ*, *AtCBF*, *AtATR1*/*MYB34*, *AtMYC2*, *AtCOR15A*, *AtRD29A* and *AtRD29B)* in *A.thaliana* overexpressing *VaPAT1*	83
*Capsicum annuum* $ \raisebox{-2pt}{\includegraphics{\bwartpath uhad220fx33}} $	*CaGRAS1*	PAT1	Improved tolerance to Drought	Modulation of stress-responsive genes (*CaLOX1* and *CaABI1)* in *CaGRAS1*-silenced pepper	84
*S. lycopersicum* $ \raisebox{-2pt}{\includegraphics{\bwartpath uhad220fx34}} $	*SlGRAS40*	HAM	Enhanced tolerance to Salinity/ Drought	Modulation of genes involved in stress responses (ROS scaveging) and hormonal signalling (auxin, gibberellin, and ethylene signalling) in *S. lycopersicum* overexpressing *SlGRAS40.*	75
*S. lycopersicum* $ \raisebox{-2pt}{\includegraphics{\bwartpath uhad220fx35}} $	*SIGRAS7*	PAT	Enhanced resistance to Salinity/ Drought	Modulation of genes involved in stress responses (ascorbate metabolism) and hormonal signalling (auxin, gibberellin, and ethylene signalling) in *S. lycopersicum* overexpressing *SlGRAS7.*	85
*S. lycopersicum* $ \raisebox{-2pt}{\includegraphics{\bwartpath uhad220fx36}} $	*SIGRAS4*	SCL9	Enhanced tolerance to Drought	Enrichment of abscisic acid (ABA)-responsive elements in *SlGRAS4* promoter; this transcription factor directly binds to *SlSnRK2.4* promoter, pivotal in ABA signalling. SlGRAS4 also activates the promoters of several antioxidant genes.	[86] [ 7],
*T. aestivum* $ \raisebox{-2pt}{\includegraphics{\bwartpath uhad220fx37}} $	*TaSCL14*	SCL	Induced tolerance to Photooxidative stress	Decreased photosynthetic capacity, and reduced tolerance to photooxidative stress in *TaSCL14*-silenced wheat	87

*PeSCL7*, from *Populus euphratica*, is salt-induced and its overexpression in transgenic *A. thaliana* and in *P. euphratica* improved tolerance to drought and salt stresses by activating enzymes involved in carbohydrate metabolism and oxidative stress mitigation [82] (Table 2, Supplementary Fig. 1). Moreover, *AtSCL7* is up-regulated under drought and salt stress conditions [15]. The castor bean (*R. communis*) gene *29 634.m002156* which presents homology to *AtSCL7* and *AtSCL4* is also induced by drought stress [39]. Interestingly, *SCL* genes *OsGRAS39* and *OsGRAS23* have also been implicated in drought tolerance in rice (Supplementary Fig. 1) due to their expression patterns under this abiotic stress [56].

On the other hand, *SlGRAS4*, a drought stress-responsive from the AtSCL9 subfamily*,* enhanced tolerance to drought stress when overexpressed in *S. lycopersicum*, while RNAi lines were hypersensitive to this stress [86]. This altered sensitivity to drought was due to the modulation of expression of a gene coding for the positive regulator of ABA signalling SlSnRK2.4. Several dehydration-induced genes involved in oxidative stress metabolism (*SlchlAPX, SlSOD, SlGPX, SlPOD,* and *SlCAT2*) were also more expressed in plants overexpressing *SlGRAS4*. Additionally, *SlGRAS4* may be also involved in cold stress tolerance based on the expression profiles [7].

Response to salinity and drought may also involve members of the HAM subfamily since *SlGRAS40* enhanced tolerance against both stresses in *S. lycopersicum* [75] (Table 2, Supplementary Fig. 1) and may play an important role in multiple abiotic stress tolerance [75]. Transgenic tomato plants overexpressing *SlGRAS40* presented phenotypes related to altered auxin and gibberellin signalling, which was suggested to stimulate DELLA accumulation under abiotic stresses, leading to enhanced antioxidative mechanisms and abiotic tolerance [75].

Similarly, in wild-type tomato plants, the *GRAS* gene *SlGRAS7*, belonging to AtPAT subfamily, was upregulated during abiotic stresses and its overexpression enhanced resistance to both drought and salt stresses (Supplementary Fig. 1). This resilience to abiotic stresses causes in overexpressing *SlGRAS7* tomato plants a delay in indicators of plant damage, such as necrosis, chlorosis, and wilting [85]. *SlGRAS7* has extremely high sequence identity with *SlGRAS12* and exhibited conserved expression patterns [7], so they may eventually have conserved functions. Moreover, the homologous genes *SlGRAS2*, *SlGRAS3*, *SlGRAS34*, and *SlGRAS7*, from AtPAT subfamily, exhibit similar expression levels responding to abiotic stress treatments (salt, cold, and heat) [7].

Still regarding the PAT1 subfamily, *GmGRAS37* was shown to be upregulated under drought and salt stresses in wild-type soybean (*Glycine max*) and its overexpression stimulated resistance to these stresses in transgenic plants [37]. According to Wang et al. [37], these functions may eventually be shared with *GmGRAS27*, *GmGRAS72*, *GmGRAS94*, and *GmGRAS115* since they belong to the same subfamily as *GmGRAS37* and share structural features. *MsGRAS51* (also PAT1 subfamily) is induced under drought stress in alfalfa [88] (Supplementary Fig. 1). Overexpression of *CaGRAS1* (PAT1 subfamily) improved drought tolerance in pepper (*Capsicum annuum*) by modulating ABA signalling but not ABA biosynthesis [84]. On the other hand, silencing of this gene led to a drought-sensitive phenotype (Supplementary Fig. 1) characterized by reduced sensitivity to ABA which disturbed stomata apertures and caused water loss [84].

Overexpressing *VaPAT1* from *Vitis amurensis* in *A. thaliana* enhanced tolerance to salinity, drought and cold; this transcription factor modulates the expression of a series of stress responsive genes [83] (Supplementary Fig. 1). Among these, were genes coding for transcription factors involved in hormonal metabolism (AtATR1/MYB34, AtMYC2), and AtSIZ1 and AtCBF1 which regulate the ICE-CBF-COR pathway involved in cold acclimation [89]. AtSIZ1 encodes a small ubiquitin-like modifier (SUMO) E3 ligase, which mediates sumoylation of ICE1, and consequently its stability leading to induced *CBF* expression and its target *COR* genes (COR15A and RD29A). These studies highlight the role of PAT1 proteins and their functional diversity under different abiotic stresses, such as drought and cold.

In another Vitis species, *Vitis vinifera*, the GRAS subfamily DELLA member *VviRGA3,* which showed a one-to-one orthologue with one gene from species, such as orange, apple, and rice, is down-regulated under salt, drought, and high light [6]. On the other hand, *VviHAM3* is up-regulated in seed and shoot tip under drought, while *VviLAS2* is up-regulated under long days and UV light [6].

Photooxidative stress is known to affect plant development and yield. The expression of *TaSCL14* in wheat (*T. aestivum L.)* was induced by high light exposure [87]. Silencing of *TaSCL14* expression caused an inhibition of plant growth along with reduction in both photosynthetic activity and tolerance to photooxidative stress [87] (Table 2, Supplementary Fig. 1).

High temperatures and carbon dioxide that can arise from climate change may affect plant development. In Korean fir (*Abies koreana*), several GRAS genes respond specifically to high CO_2_ stress [90]. Heat stress led to the up-regulation of *SCL13,* a member of the PAT1 sub-branch, in *A. thaliana,* but only during the early hours of the day, suggesting a connection between the circadian clock and heat stress response regulators [91].

So far, studies involving transcriptional profiling and functional analyses of GRAS genes conducted in model and crop plants indicate that abiotic stress responses involve mainly members from SCL, PAT, and HAM subfamilies that lead to modulation of oxidative stress and hormonal metabolism to promote survival under stress.

**Table 3:** Mechanisms involving GRAS genes in response to multiple biotic stresses as revealed by functional analyses

Species	GRAS gene	Function	Target genes/Pathways	Reference
*Arabidopsis thaliana* $ \raisebox{-2pt}{\includegraphics{\bwartpath uhad220fx38}} $	*AtRGL3*	Resistance against *Botrytis cinerea* and A*lternaria brassicicola.* Susceptibility against *Pseudomonas syringae* pv. *tomato* strain DC3000.	The control of SA pathway is achieved through interaction of C-terminus of RGL3 GRAS domain and the N-terminus of EDS1 lipase-like domain leading to a decrease of SA perception. Positive regulation of JA signalling occurs in a MYC2-dependent manner: at promotor level, MYC2 enhances RGL3 accumulation favoring the sequestration of JAZ1 repressor and activation of downstream JA-responsive genes.	[87,94,97]
*A. thaliana* $ \raisebox{-2pt}{\includegraphics{\bwartpath uhad220fx39}} $	*AtRGA*	Tolerance against *P. syringae* pv. *tomato* strain DC3000 after elicitation with *Xanthomonas campestris* effector XopD_*Xcc*8004_.	Delay on GA-induced degradation of RGA though interaction between the N-terminal DELLA regulatory domain of RGA and N-terminal ERF-associated amphiphilic repression (EAR) motif region of XopD_*Xcc*8004_ protein leading to an interference with the ligation to the GA-receptor GID1.	[100]
*Oryza sativa* $ \raisebox{-2pt}{\includegraphics{\bwartpath uhad220fx40}} $	*OsSLR1*	Resistance against *Magnaporthe oryzae* and *Xanthomonas oryzae* pv. *oryzae.*	Negative control of GA-mediated responses and positive regulation and amplification of the cooperative interaction between SA/JAs signalling pathways.	[99]
*O. sativa* $ \raisebox{-2pt}{\includegraphics{\bwartpath uhad220fx41}} $	*OsCIGR1–2*	Induction after elicitation with the effector N-acetyl-chitooligosacharide.	*OsCIGR2* integrates the resistance mechanisms against *Magnaporthe oryzae* through interaction with the B-type heat shock OsHsf23 and avoidance of excessive cell death.	[105,101]
*Manihot esculenta* $ \raisebox{-2pt}{\includegraphics{\bwartpath uhad220fx42}} $	*MeDELLA1–4*	Induction after elicitation with flagellin 22. Involvement in disease resistance against *Xanthomonas axonopodis* pv. *manihotis*.	Involvement in early defence responses due to the activation of PAMP-triggered immunity through positive regulation of callose accumulation and trigger of pathogenesis-related genes (*MePR1–4*) expression.	[101]
*Raphanus sativus L. var radiculus* $ \raisebox{-2pt}{\includegraphics{\bwartpath uhad220fx43}} $	*RsRGA*	Tolerance against *X. campestris* pv. *campestris* after elicitation with *X. campestris* effector XopD_*Xcc*8004_*.*	Delay on GA-induced degradation of RGA though interaction between the N-terminal DELLA regulatory domain of RGA and N-terminal ERF-associated amphiphilic repression (EAR) motif region of XopD_*Xcc*8004_ protein leading to an interference with the ligation to the GA-receptor GID1.	[100]
*Solanum lycopersicum* $ \raisebox{-2pt}{\includegraphics{\bwartpath uhad220fx44}} $	*SlGRAS1–4, SlGRAS6, SlGRAS13*	Induction upon infection with *P. syringae* pv. *tomato* strain DC3000.	Modulation of *SlGRAS1–3/SlGRAS6* (SlPAT1 subfamily) and *SlGRAS4/SlGRAS13* (SlSCL9 subfamily) expression upon *Pst*DC300 infection. *SlGRAS6* confers complete resistance against *Pst*DC300 and might act as a downstream element on Pto/AvrPto signalling events.	[103,104]

**Figure 3:**
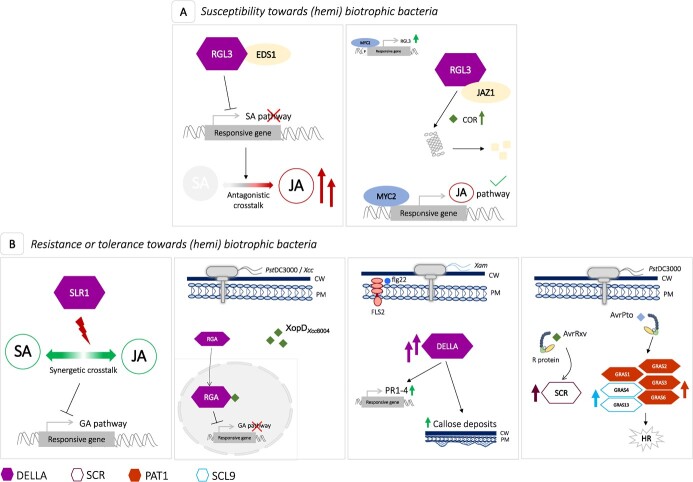
General model for the involvement of GRAS subfamilies in host defence responses towards (hemi) biotrophic bacteria pathogens. **(A)** Stabilization of RGL3 – EDS1 interaction leads to a decrease of SA perception (likely through inhibition of SA biosynthesis and/or signalling) or through the synthesis of COR phytotoxin, which mimics JA to enhance the JA-mediated responses. In a JA-dependent manner, RGL3 sequesters JAZ1 and stimulates JAZ1 degradation at the proteosome. Then, free MYC2, a transcriptional activator, induces RGL3 and JA-responsive genes ultimately leading to susceptibility. **(B)** Resistance results from SLR1 integration as a positive regulator of defense responses by interfering with GA signalling and favoring and amplifying a cooperative relation between SA and JA pathways; or when the effector XopD_*Xcc*8004_ acts as a promotor of disease tolerance against *Pseudomonas syringae* pv. *tomato* (*Pst*) strain DC3000 (*Pst*DC3000) and *Xanthomonas campestris* pv. *campestris* (*Xcc*) by targeting and partially stabilizing RGA at the nucleus which will repress temporally GA-mediated degradation of RGA; also, infection with *Xanthomonas axonopodis* pv. *manihotis* (*Xam*) or pathogen recognition by host receptors (e.g. through FLS2) triggers DELLA-mediated responses and induces defense-related genes (e.g. *PR1–4*) and the deposition of callose on the cell wall; finally, recognition of *X. campestris* pv. *vesicatoria* (*Xcv*) effectors AvrRxv and *Pst*DC300 effector AvrPto leads to R-mediated defense responses triggering three subfamilies of GRAS, namely SCR, PAT1 and SCL9 subfamily. Abbreviations: COR, Coronatine; CW, Cell wall; EDS1, Enhanced disease susceptibility 1; flg22, flagellin 22; FLS2, Flagellin sensitive2 receptor; GA, Gibberellins; HR, Hypersensitive response; JA, Jasmonate; JAZ1, Jasmoante ZIM-domain 1; MYC2, bHLHzip transcription factor MYC2; PR, Pathogen related protein; RGA, *REPRESSOR OF GA1*; RGL3, DELLA RGA-LIKE3; SA, Salicylic acid.

### Involvement of GRAS in biotic stress responses

GRAS family controls a plethora of signal transduction pathways related to resilience toward biotic stresses, and several mechanistic insights have been explored in this regard. Against bacteria pathogens, DELLAs are predominantly described as central hubs of defence responses [6] and have been associated with the susceptibility of Arabidopsis against the hemibiotrophic *Pseudomonas syringae* pv. *tomato* (*Pst*). Li et al. [92] revealed an interaction between DELLA RGA-LIKE3 (RGL3) and EDS1, an upstream element of the salicylic acid (SA) pathway [93] to decrease SA perception (Table 3; Fig. 3, A). In accordance, Arabidopsis quadruple-DELLA mutants (*gai*, *rga*, *rgl1*, *rgl2*) challenged with this bacteria exhibited higher levels of SA, signs of hypersensitive response and delayed expression of genes involved in the JA metabolism [94]. The outcome of a plant–pathogen interaction is tightly controlled by an hormonal blend [95]; a classical view relates SA and jasmonic acid (JA) with resistance against biotrophic and necrotrophic pathogens, respectively [96]. The bacterium *Pst*DC3000 synthesizes the phytotoxin coronatine (COR), which mimics JA (Table 3; Fig. 3, A) [94, 97]. In a COR-dependent manner, responsiveness to JA is given by RGL3 sequestration of JAZ1 at the nucleus, which leads to the release of the transcriptional activator of JA metabolism MYC2 and induction of JA-responsive genes [97, 98]. Interestingly and contrary to what was described in Arabidopsis, the rice DELLAs Slender Rice 1 (SLR1) boosted the basal immunity and acted as positive regulator against hemibiotrophics by interfering with GA signalling, favouring SA/JAs pathways and even amplifying their mediated signalling (Table 3; Fig. 3, B) [99]. In Arabidopsis and radish, challenged with bacteria *Xanthomonas campestris* effector XopD_*Xcc*8004_, was noticed induction of DELLAs RGA-mediated responses against *Pst*DC3000 and *X. campestris* pv. *campestris* (*Xcc*), respectively [100]. Targeting of the effector to RGA and partial stabilization at the nucleus resulted in temporal repression of GA signal transduction and delay of disease symptoms [100] (Table 3; Fig. 3, B). Cassava infection with the causal agent of bacterial blight or, after elicitation with the bacterial effector flagellin 22 (flg22), increased the expression of *MeDELLA1–4* genes and defence-related genes (Table 3; Fig. 3, B) [101]. Besides *DELLAs*, other *GRAS* have been associated with transcriptome reprogramming in response to bacterial infection. In tomato challenged with the *X. campestris* effector AvrRxv, this reprogramming included two *SCR* genes [102]. During the incompatible interaction with the bacteria *Pst*DC3000, Mayrose et al. and Mysore et al. [98, 99] notice the responsiveness of tomato genes belonging to SlPAT1 (*SlGRAS1–3*, *SlGRAS6*) and SlSCL9 (*SlGRAS4*, *SlGRAS13*) subfamilies. *SlGRAS6* also responded to *Xcv* infection and was shown to be involved in complete resistance against *Pst*DC3000 (Table 3; Fig. 3, B) [103].

**Figure 4:**
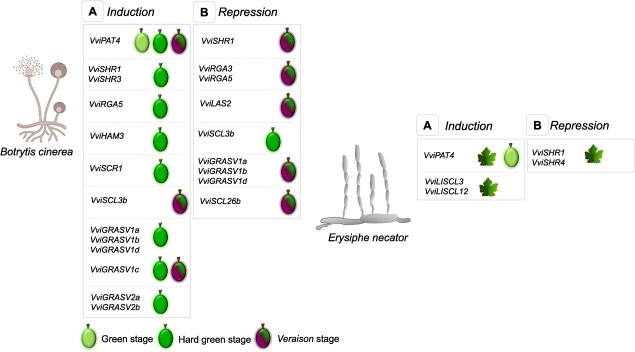
Involvement of GRAS subfamilies in grapevine defence responses towards the necrotrophic fungus *Botrytis cinerea* (causal agent of Grey mould) and the biotrophic fungus *Erysiphe necator* (causal agent of Powdery mildew). Regarding the interaction with *B. cinerea*, RNAseq data was obtained from grape berries at green (EL32), hard green (EL33) and *veraison* (EL35) ripening stages from the susceptible cultivar *Vitis vinifera* cv. Trincadeira. Regarding the interaction with *E. necator*, RNAseq data was obtained from leaves of susceptible *V. vinifera* cv. Cabernet Sauvignon and hard green (EL33) grape berries from susceptible *V. vinifera* cv. Carignan.

In the context of fungal infections, no GRAS subfamily appears to be the central coordinator of defence responses, but features can be shared with other pathogens [6]. Against the necrotrophic *Alternaria brassicicola*, *RGL3*, the most responsive *DELLA* gene to biotic stress, plays an essential role (Table 3) [94]. Upon infection with the necrotrophic *Botrytis cinerea*, JA metabolism of Arabidopsis was also activated in an RGL3-dependent manner [94, 97]. The GRAS subfamilies VviSCR (e.g. *VviSCR1*), VviSCL3 (e.g. *VviSCL3b*), VviGRASV2 (e.g., *VviGRASV2a-b*), VviSCL26 (e.g. *VviSCL26b*), and VviGRASV1 (e.g. *VviGRASV1a-d*), seem to be specific to defence responses against *B. cinerea* in grapevine (Fig. 4) [6]. Additionally, the expression of genes from the VviRGA subfamily was shown to be genotype- and developmental stage-specific during this interaction (Fig. 4) [106, 107]. For grapevine leafroll-associated virus 3 (GLRaV-3) involvement of *VviRGA3* was also observed [108]. In a susceptible grapevine species, infection with *B. cinerea* modulated diverse GRAS subfamilies with the majority being positively regulated at an earlier stage of interaction when defensive response is stronger (Fig. 4) [106]. *VviPAT4* was induced in susceptible species infected with *B. cinerea* and also with *Erysiphe necator* (causal agent of Powdery mildew) and Bois noir phytoplasma [109–111]. The response of *VviPAT4* to UV exposure, salinity, drought, and cold suggest a common integration in biotic and abiotic stresses and, as for its orthologous *SlGRAS2* in tomato, might be due to its involvement in the hormonal network [6, 7]. In fact, cold tolerance in Arabidopsis was achieved in a JA-dependent manner through positive regulation of *VaPAT1* from *V. amurensis* [112]. Regarding the VviSHR subfamily, *VviSHR1* and *VviSHR3* were associated with response against *B. cinerea* (Fig. 4) [6]. Interestingly, *VviSHR3* co-expressed with *stilbene synthase* gene [*113**]* involved in the synthesis of defensive stilbenoids. *VviSHR1* and *VviSHR4* were the only responsive genes upon PM [110]. Upon GLRaV-3 and Bois noir, *VviSHR1* was also induced [108, 109]. *SHR1* is conserved among species and is co-expressed with genes mostly involved in cell wall degradation and signalling [6] which occurs under infection [114]. Most VviLISCL subfamily genes showed distinctive expression patterns in response to *B. cinerea* infection suggesting a functional specialization (Fig. 4). Expression of *VviLISCL12* and *VviLISCL3* increased in susceptible leaves upon PM, Bois noir, and GLRaV-3 (Fig. 4) [108–110]. These genes are homologous of *AtSCL14*. AtSCL14 acts as a transcriptional co-activator of genes involved in broad-spectrum detoxification networks and might be used to maintain the balance of reactive species [115, 116]. Regarding VviLAS subfamily, *VviLAS2* was only detected in susceptible species under *B. cinerea* infection (Fig. 4) [106]. This gene was also expressed in susceptible GLRaV3-infected leaves and co-expressed with genes involved in biotic responses (e.g. epoxide hydrolases) [6]. The expression of *VviHAM3* increased in grapes and leaves infected with *B. cinerea*, Bois noir, GLRaV-3 (Fig. 4) [106, 108, 109]. In *B. distachyon*, members of the HAM subfamily, had their expression increased significantly after infection with *M. oryzea* [55]. Two rice genes included in the *AtPAT1* sub-branch, chitin-inducible gibberellin-responsive 1 (Os*CIGR1)* and Os*CIGR2*, were activated in cell-suspension after perception of the effector N-acetyl-chitooligosaccharide revealing an involvement in early signalling responses (Table 3) [83, 117]. Eventually, due to a functional specialization towards fungi attack in rice, OsCIGR2 induced the expression of the B-type heat shock *OsHsf23*, which helped to control excessive cell death during infection and avoided the perpetuation of *M. oryzae* biotrophy (Table 3) [105].

*GRAS* genes are also involved in arbuscular mycorrhiza development. Gibberellin metabolism is fine-tuned by a complex regulatory mechanism controlled by DELLA, but where other GRAS proteins, like Nodulation signalling pathway 1 (NPS1) and NPS2 are also involved [118, 119]. The GRAS Required for Arbuscule Development 1 (RAD1) is particularly essential for arbuscular mycorrhiza development [120]. However, in *M. truncatula*, RAD1-mediated responses facilitated the root colonization by the pathogenic oomycetes *Phytophthora palmivora* [*121**,**122**]*. Two *SCL* genes in Arabidopsis, *AtSCL6* and *AtSCL21* were targeted of secreted parasitic proteins from the root-knot nematode *Meloidogyne incognita* to increase the success of its life cycle [123]. Altogether, GRAS families have a multifaceted role since they are triggered by a wide range of pathogenic species and can be associated with susceptibility or resistance defensive related responses, as well as participating in beneficial plant–fungus interactions.

### GRAS transcription factors and crop improvement

GRAS genes have great potential as targets for crop improvement due to their wide involvement in plant growth and development and stress responses [124, 125]. This can be achieved by gene editing methods among which CRISPR has been more widely adopted in recent years and is currently being optimized at an increasingly rapid pace [126]. This technology can be used for exploring gene function and as a modern breeding technique for development of new plant varieties with important traits, such as high nutritional value, high yield, and resistance to biotic and abiotic stresses that are likely to become more problematic in a near future due to climate change [127].

The GRAS protein DELLA is able to interact with proteins and affect phytohormone signalling pathways, which can be used as a starting point for improve crop breeding strategies [127]. Amino acid substitutions and deletions in the DELLA domain of GA-insensitive (GAI) using the CRISPR/Cas system resulted in gibberellic acid-induced susceptibility to degradation, causing a dwarf phenotype in *A. thaliana* [128]. This dwarf phenotype is caused because gibberellic promotes degradation of DELLA proteins, which are negative growth regulators [129], therefore, the amino acid substitutions and deletions in the DELLA domain of GAI decreased the degradation of DELLA protein and affected plant development. Similarly in tomato, loss-of-function mutations obtained by CRISPR on PROCERA, a tomato gene that encodes a DELLA protein, resulted in derepressed growth [129]. In addition, the use of the CRISPR/Cas9 system to cause a loss-of-function mutation of the rice *SLR1* gene, that encodes the DELLA protein, generated a dwarf phenotype by inhibiting plant growth [130]. All these dwarf phenotypes reduced plant height and compact growth habits. In grapevine, internodes of the GAI1 mutant microvine are five time shorter that normal phenotype leading to shorter plants in the same growth period [131]. This could be useful in the cultivation of plants where the use of special machinery for the treatment of plants, pruning and management of stems and branches, may be required.

All these functional studies on GRAS transcription factors highlight how gene editing may be powerful in generating new crop varieties. However, for speeding up the applications of these technologies in crop improvement development of a product-based regulatory policy on genome edited plants is critical [132]. Under the current European Union legislation, crop varieties obtained by using technologies, such as CRISPR/Cas9, are no longer subjected to strict genetically modified organism regulations but the legislation still does not offer the adequate framework for wide investment in these varieties [133].

Another challenge is the targeting of duplicated genes, which can present high co-expression across tissues and therefore exhibit a certain level of gene redundancy [6]. Many of the GRAS genes have redundant copies and functional studies often need multiple mutants to study their role. However, these GRAS genes can also enable the achievement of important commercial phenotypes, such as the slowing down of fruit ripening but mutating one of the duplicated genes whose expression is higher in ripe fruit. This represents tremendous opportunity in postharvest research given that fruit quality may not be affected. Moreover, the hereby explored duplicate gene retention is an intriguing subject in evo–devo modelling [134]. The contributions of gene duplication to gene regulatory networks, and adaptive evolution are still a matter of debate. In this context, it can also have occurred that specific GRAS genes involved in stress resilience were lost during crop domestication. Transcription factors indeed play a central role in the process of crop domestication [135] and retrieving these lost genes by exploring genomes from crop wild relatives through *de novo* domestication may also open exciting avenues for crop improvement [136]. In fact, recent analyses indicate that transcription factors presented higher rates of molecular evolution than their structural gene targets in the biochemical pathways they regulate [137].

## Conclusions and future perspectives

The first member of the GRAS domain family (SCARECROW-SCR) was identified in 1996 [51]. Since then, diverse studies have been carried out on this plant gene family. In more than two decades *in silico* and functional analyses have been conducted in several monocot and dicot species as explored in this review. Genome sequencing data have been rapidly accumulated in crops enabling functional characterization in these species besides model plants.

GRAS family of transcription factors is as fascinating as challenging. In fact, no subfamily is solely associated with a particular role and even the same gene can present opposite response when submitted to different stresses highlighting their functional diversity. Nevertheless, the role played by many previously and recently identified subfamilies in growth, development and stress responses of diverse plant species is still unknown, leaving room for further functional studies. So far DELLA subfamily appears to be extremely versatile by being involved in several of these processes. Some ancestral gene regulatory networks also seem highly conserved such is the case of involvement of HAM subfamily in shoot apical meristem maintenance suggesting that the role of GRAS associated with developmental regulation may eventually be more conserved that with environmental regulation.

It is also clear that GRAS orthologues may assume shared or different biological functions in different plant species. We also explored in this review that though duplicated genes may retain similar functions, phylogenetic analysis of GRAS genes may also present limitations to infer the functions of uncharacterized GRAS members based on their evolutionary history and sequence similarity. A crucial issue is also the chaotic nomenclature reported in the literature. In this context, agreement of nomenclature rules for GRAS and other transcription factors and even the launching of a specific database for GRAS would speed up comparative genetics/genomics and functional studies. It would be particularly interesting to include in this database GRAS target genes using DAP-seq for their identification. This would greatly contribute to clarify the molecular networks involving GRAS in plant development and stress resilience and therefore, generate new crops to cope with climate change.

## Acknowledgements

Fundação para a Ciência e Tecnologia (FCT) supported the research through Vinisense project (PTDC/BAA-DIG/4735/2020) and Research Unit grant UID/MULTI/04046/2021, awarded to BioISI. R.A. is a recipient of fellowship from BioSys PhD programme PD65-2012 (UI/BD/153054/2022). We deeply thank Dr. Pedro Humberto Castro (CIBIO) for critically revising the manuscript.

## Author Contribution Statement

AMF conceived the review. C. N., B. R., R. A., J. E., and A.M.F. wrote the review. J. G. critically revised and edited the review.

## Conflict of interests Statement

The authors declare the absence of conflict of interest.

## Supplementary Data

Supplementary data is available at *Horticulture Research* online.

## Supplementary Material

Web_Material_uhad220
