# Supplementary material for: Network of GRAS transcription factors in plant development, fruit ripening and stress responses
Source: Hortic Res. 2023 Sep 27;10(12):uhad220. doi: 10.1093/hr/uhad220 (PMC10699852; doi:10.1093/hr/uhad220)

Supplementary Table S1. Number and characteristics of GRAS genes found in different plant species. The genome size was retrieved from https://www.ncbi.nlm.nih.gov/.

Species	Genome size (bp)	Number of GRAS genes	Gene length (bp)	Protein length (aa)	Percentage of intronless genes	Number of subfamilies	References
Arabidopsis thaliana 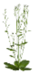	119 668 634	34	-	202-769	67,60%	10	(X. Liu & Widmer, 2014)
	119 668 634	32	-	371-1323	-	8	(Tian et al., 2004)
	119 668 634	33	-	-	90,90%	10	(B. Zhang et al., 2018)
	119 668 634	33	-	-	-	13	(Cenci & Rouard, 2017)
Populus trichocarpa 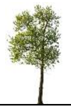	392 162 179	106	-	79-1263	54,70%	13	(X. Liu & Widmer, 2014)
Oryza sativa 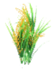	374 422 835	60	-	137-977	55%	12	(X. Liu & Widmer, 2014)
	374 422 835	57	-	276-977	-	8	(Tian et al., 2004)
	374 422 835	50	-	-	80%	13	(B. Zhang et al., 2018)
	374 422 835	56	-	-	-	16	(Cenci & Rouard, 2017)
Cucumis melo 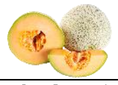	438 408 223	37	1445-4234	-	59,40%	9	(Bi et al., 2021)
Malus domestica 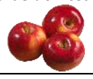	703 358 299	127	333-5787	110-1928	55,12%	8	(Fan et al., 2017)
Vitis vinifera 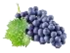	486 196 837	52	294-2349	-	88,46%	13	(Grimplet et al., 2016)
	486 196 837	49	-	-	-	17	(Cenci & Rouard, 2017)
Brassica napus 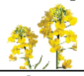	1 001 886 053	87	-	404-745	89,70%	13	(Guo et al., 2019)
Solanum lycopersicum 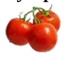	824 010 332	53	-	125-864	77,40%	13	(Huang et al., 2015)
Capsicum annuum 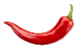	3 212 488 018	50	-	419-801	84%	10	(B. Liu et al., 2018)
Triticum aestivum 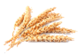	14 566 954 962	188	-	245-819	60%	12	(Yanfeng Liu & Wang, 2021)
Brachypodium distachyon 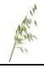	271 298 618	48	-	150-805	85,42%	10	(Niu et al., 2019)

Lagenaria siceraria 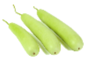	297 879 142	37	-	378-1466	67,57%	16	(Sidhu et al., 2020)
Hordeum vulgare 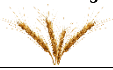	4 225 713 981	62	-	121-792	74,20%	12	(To et al., 2020)
Solanum tuberosum 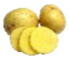	705 934 411	52	-	350-800	90%	8	(S. Wang et al., 2019)
Glycine max 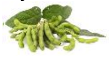	978 941 695	117	510-2532	169-843	77,78%	9	(L. Wang et al., 2021)
	978 941 695	117	-	168-842	80,34%	11	(T. T. Wang et al., 2020)
Panax ginseng 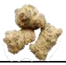	3 355 148 547	59	201-3343	30-798	-	13	(N. Wang et al., 2020)
Ricinus communis 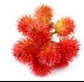	315 619 833	48	1008-2532	335-843	78,30%	13	(Xu et al., 2016)
Dendrobium catenatum 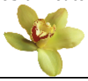	1 104 259 548	47	-	345-757	82,90%	11	(Zeng et al., 2019)
Gossypium hirsutum 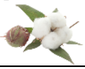	2 306 070 423	150	-	208-1821	82%	14	(B. Zhang et al., 2018)
Gossypium arboreum 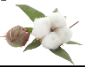	1 694 597 732	77	-	-	79,22%	13	(B. Zhang et al., 2018)
Gossypium raimondii 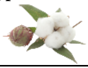	761 251 514	82	-	-	73,17%	13	(B. Zhang et al., 2018)
Physcomitrium patens 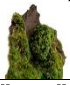	472 081 022	42	-	-	35,71%	15	(B. Zhang et al., 2018)
Selaginella moellendorffii 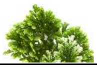	212 315 224	46	-	-	84,78%	15	(B. Zhang et al., 2018)
Amborella trichopoda 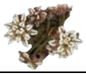	706 495 326	34	-	-	-	17	(Cenci & Rouard, 2017)
Phoenix dactylifera 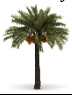	773 189 301	59	-	-	-	17	(Cenci & Rouard, 2017)
Musa acuminata 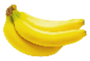	472 231 289	72	-	-	-	16	(Cenci & Rouard, 2017)
Theobroma cacao 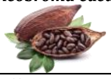	324 879 930	44	-	-	-	17	(Cenci & Rouard, 2017)
Coffea canephora 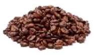	568 611 505	50	-	-	-	17	(Cenci & Rouard, 2017)
Fragaria vesca 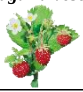	214 373 013	54	1320-4904	375-836	-	14	(Chen et al., 2019)
Prunus mume 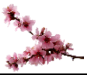	234 030 239	46	-	392-941	82,61%	11	(Lu et al., 2015)

Supplementary Figure 1. Involvement of GRAS genes and corresponding subfamilies in response to abiotic stress (drought, salinity, photooxidative stress and cold). Species where the studies were conducted are shown in parenthesis: *At: Arabidopsis thaliana, Os: Oryza sativa, Sl: Solanum lycopersicum, Ms: Medicago sativa, Ta: Triticum avestum.* References were mentioned in the text.

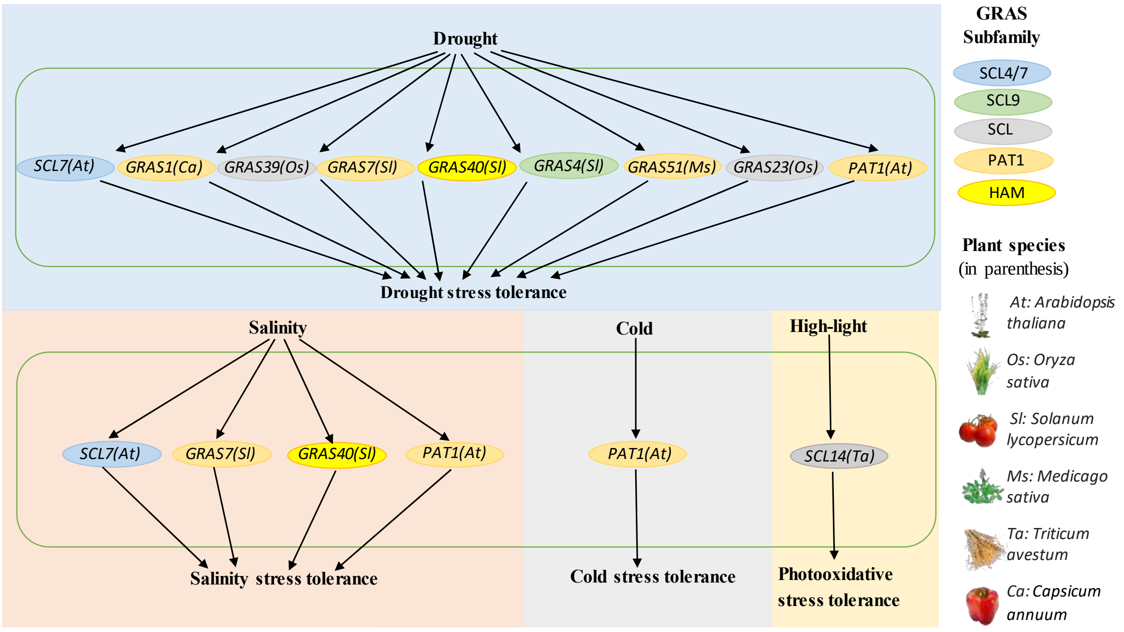

Supplement: Web_Material_uhad220 [file web_material_uhad220.pdf]
